# Stargardt disease-associated in-frame *ABCA4* exon 17 skipping results in significant ABCA4 function

**DOI:** 10.1186/s12967-023-04406-x

**Published:** 2023-08-16

**Authors:** Melita Kaltak, Rocio Blanco-Garavito, Laurie L. Molday, Claire-Marie Dhaenens, Eric E. Souied, Gerard Platenburg, Jim Swildens, Robert S. Molday, Frans P. M. Cremers

**Affiliations:** 1 Department of Human Genetics, Radboud University Medical Center, Nijmegen, The Netherlands; 2 ProQR Therapeutics, Leiden, The Netherlands; 3 Department of Ophthalmology, Intercommunal Hospital Center and Henri Mondor Hospital, Paris-Est Créteil University, Creteil, France; 4 Department of Biochemistry and Molecular Biology, Department of Ophthalmology and Visual Sciences, Centre for Macular Research, University of British Columbia, Vancouver, BC Canada; 5 University of Lille, Inserm, CHU Lille, U1172-LilNCog-Lille Neuroscience & Cognition, Lille, France

## Abstract

**Background:**

*ABCA4*, the gene implicated in Stargardt disease (STGD1), contains 50 exons, of which 17 contain multiples of three nucleotides. The impact of in-frame exon skipping is yet to be determined. Antisense oligonucleotides (AONs) have been investigated in Usher syndrome-associated genes to induce skipping of in-frame exons carrying severe variants and mitigate their disease-linked effect. Upon the identification of a STGD1 proband carrying a novel exon 17 canonical splice site variant, the activity of ABCA4 lacking 22 amino acids encoded by exon 17 was examined, followed by design of AONs able to induce exon 17 skipping.

**Methods:**

A STGD1 proband was compound heterozygous for the splice variant c.2653+1G>A, that was predicted to result in in-frame skipping of exon 17, and a null variant [c.735T>G, p.(Tyr245*)]. Clinical characteristics of this proband were studied using multi-modal imaging and complete ophthalmological examination. The aberrant splicing of c.2653+1G>A was investigated in vitro in HEK293T cells with wild-type and mutant midigenes. The residual activity of the mutant ABCA4 protein lacking Asp864-Gly885 encoded by exon 17 was analyzed with all-trans-retinal-activated ATPase activity assay, along with its subcellular localization. To induce exon 17 skipping, the effect of 40 AONs was examined in vitro in WT WERI-Rb-1 cells and 3D human retinal organoids.

**Results:**

Late onset STGD1 in the proband suggests that c.2653+1G>A does not have a fully deleterious effect. The in vitro splice assay confirmed that this variant leads to *ABCA4* transcripts without exon 17. ABCA4 Asp864_Gly863del was stable and retained 58% all-trans-retinal-activated ATPase activity compared to WT ABCA4. This sequence is located in an unstructured linker region between transmembrane domain 6 and nucleotide-binding domain-1 of ABCA4. AONs were designed to possibly reduce pathogenicity of severe variants harbored in exon 17. The best AON achieved 59% of exon 17 skipping in retinal organoids.

**Conclusions:**

Exon 17 deletion in *ABCA4* does not result in the absence of protein activity and does not cause a severe STGD1 phenotype when in *trans* with a null allele. By applying AONs, the effect of severe variants in exon 17 can potentially be ameliorated by exon skipping, thus generating partial ABCA4 activity in STGD1 patients.

**Graphical abstract:**

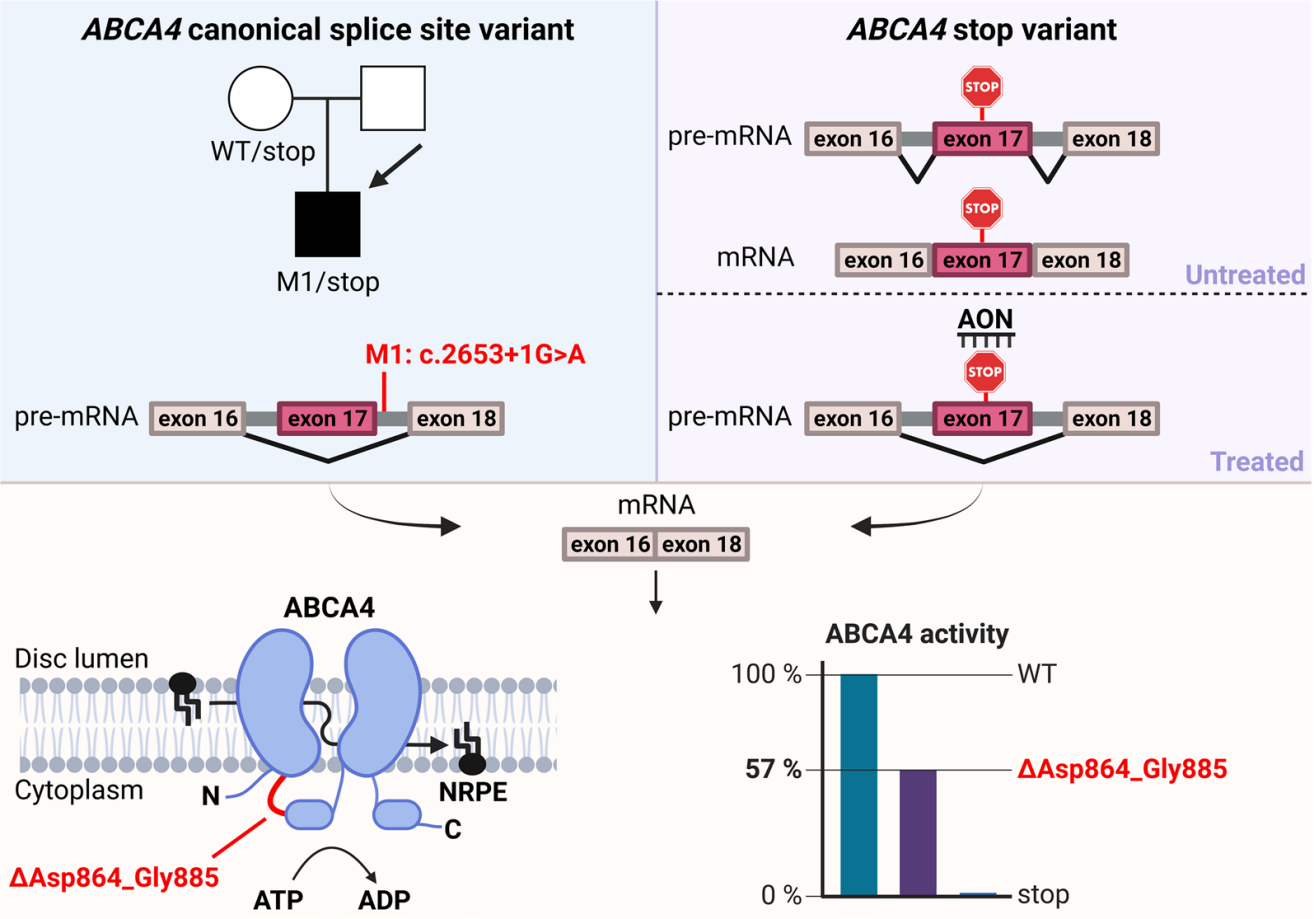

**Supplementary Information:**

The online version contains supplementary material available at 10.1186/s12967-023-04406-x.

## Introduction

Biallelic variants in the *ABCA4* gene are implicated in Stargardt disease type 1 (STGD1) [1], characterized by progressive macular degeneration, fundus flecks caused by amassed toxic metabolites formed during the visual cycle and a peripapillary region resistant to disease-associated changes [2]. Onset can be in the first decade of life which has been described as a panretinal cone-rod dystrophy [3, 4]. More recently, a better appreciation of the role of mild or hypomorphic variants, when in *trans* with severe or null alleles, has revealed an increasing number of persons with late-onset STGD1 [5–7]. STGD1 is considered the most prevalent form of inherited macular dystrophy and it is estimated that there are 1,385,000 affected individuals worldwide [8]. Interestingly, some of the mild variants have been shown, or are suspected, to be incompletely penetrant, when in *trans* with a severe or null allele. In addition, a sex imbalance (more females than males affected) was observed for probands carrying c.5603A>T, p.(Asn1868Ile) or c.5882G>A, p.(Gly1961Glu), in *trans* with a severe or null allele [5, 6]. These results suggest that a significant part of STGD1 probands are subject to polygenic or multifactorial inheritance in which biallelic *ABCA4* variants are needed but not enough to develop STGD1.

The structure of ABCA4 was reported to consist of two transmembrane domains (TMD), each consisting of 6 helical membrane spanning segments, two glycosylated exocytoplasmic domains (ECD) and two nucleotide-binding domains (NBD) divided over two non-symmetric consecutive halves [9, 10]. The cryo-EM structure of the 2273 amino acid transporter has been previously reported in its substrate-bound, nucleotide-bound and unbound states [11–13]. These studies gave insight in its size and suggested that the ABCA4 localization in the rims of photoreceptors’ outer segment disk membranes is predetermined because of its elongated ECDs which do not fit in the flattened parts of the photoreceptor disks. ABCA4 plays a critical role in the visual cycle by acting as a flippase for retinoids, which limits the accumulation of toxic bisretinoid compounds responsible for retinal degeneration [14].

To date, STGD1 remains an untreatable condition. Even though several clinical trials are ongoing (NCT03033108, NCT05244304, NCT03364153, NCT04545736), none of the investigated therapeutic approaches tackles the molecular genetic defects underlying the disease but is rather focused on managing the toxic vitamin-A metabolites or suppressing the complement component 5 in the complement system to reduce inflammation in the retinal pigment epithelium upon accumulation of A2E and bisretinoids [15]. Based on the variants’ allele numbers reported by Cornelis et al. in biallelic probands [16], 19% of 2167 unique variants and 23% of all 11,146 disease-associated alleles, affect splicing. The high incidence of splicing variants, of which many result in the insertion of pseudoexons in the mRNA, make STGD1 a great target for antisense oligonucleotide (AON)-based therapies.

Moreover, AONs can be used to redirect the splicing and skip coding regions without interfering with the translation of the rest of the protein [17]. AONs were firstly used to suppress expression of several disease-causing genes [18–22], with formivirsen pioneering as the first FDA-approved AON for treatment of cytomegalovirus retinitis [23]. Splicing modulating AON-based therapies were introduced in 2016 with Sarepta Therapeutics’ eteplirsen and, later on, golodirsen, which excluded ‘in-frame’ exons 51 or 53 in *DMD*, respectively. The open reading frame was maintained and the severe phenotype of Duchenne muscular dystrophy (DMD) due to protein-truncating variants in these exons was ameliorated [24, 25].

AON-based skipping of a coding region was also previously investigated for *USH2A*-associated retinitis pigmentosa (RP) and Usher syndrome (USH) variants that result in a premature stop of translation or in damaging amino acid substitutions [26–28]. The clinical trials focused on probands carrying the frequent *USH2A* exon 13 variants c.2299delG, p.(Glu767Serfs*21) and c.2276G>T, p.(Cys759Phe) [26].

In *ABCA4*, AONs were applied to either exclude pathogenic intronic insertions (a.k.a. pseudoexons) or restore wild-type splicing of exons 39 and 40 due to c.5461-10T>C [29–35]. Therapeutic intervention through the exclusion of exons carrying pathogenic variants was not yet considered as the above-described structure of the protein suggested that the complete coding region was needed for a correct function of ABCA4. *ABCA4*, consisting of 50 protein-coding exons, contains 17 exons consisting of a multiple of three nucleotides, which, if skipped, hypothetically, would not disrupt the open reading frame and a shorter yet potentially functional protein would be translated. In-frame skipping of nonessential exons could be a therapeutic approach for alleviating the effect of severe missense variants in *ABCA4*, however, the effect of in-frame exon skipping is unknown.

In this study, we report the first evidence of in-frame exon skipping that results in significant activity of ABCA4. We identified that the 22 amino acids encoded by exon 17, which are located entirely in a segment connecting TMD1 and NBD1 in ABCA4, are not crucial for the protein’s function. Following these findings, we developed an AON-based intervention designed to skip exon 17 that could potentially be used to treat severe variants harbored in this exon and increase the residual activity of ABCA4 in STGD1 patients.

## Materials and methods

### Clinical analysis

Clinical examination was performed with standard slit lamp and indirect ophthalmoscopy. Visual acuity was measured using ETDR charts. Fundus autofluorescence (FAF), spectral domain optical coherence tomography (SD-OCT) and fluorescein angiography (FA) were performed using Spectralis (HRA, Heidelberg Engineering, Heidelberg, Germany). Full field electroretinography (ffERG) was performed using a Ganzfeld bowl with standard ISCEV protocol (MonPackOne, Metrovision, Perenchies, France). Normative population data was used for comparison of the results for electrophysiology.

### Construction and characterization of the *ABCA4* c.2653+1G>A midigene

The BA13 construct spanning *ABCA4* exons 16, 17 and 18, and 591 bp of upstream and 1211 bp of downstream intronic sequences, was used as WT template for generating the *ABCA4* c.2653+1G> A midigene [36]. The variant was inserted by mutagenesis using the following primer sequences: 5′-ttggcttggcggtgaagatgagtcctttaaaacac-3′ and 5′-gtgttttaaaggactcatcttcaccgccaagccaa-3′. The mutated *ABCA4* sequence was placed in the pCI NEO vector [37] by the Gateway cloning system as described previously [38]. The construct was linearized with *Sal*I (New England Biolabs, Ipswich, MA, USA) and its sequence was validated with PacBio sequencing.

HEK293T cells were transfected with the mutant and WT constructs in 12-well plates at a concentration of 100 ng/well. The cells were harvested 48 h later and their RNA was extracted, and reverse-transcribed in cDNA as reported elsewhere [38]. To assess the isoform content of the cDNA, the forward primer binding in *ABCA4* exon 16 (5′-agatggaagccatgttggag-3′) and the reverse primer annealing to exon 18 (5′-tggaatctcttgaccagcag-3′) were used in RT-PCR as described previously [36]. The sequences of the identified isoforms were determined with Sanger sequencing using the above-mentioned forward primer in *ABCA4* exon 16.

### Construction, localization and functionality assessment of ABCA4 ΔAsp864_Gly885

The WT *ABCA4* coding sequence was introduced in the pcDNA™3.1 ( + ) Mammalian Expression Vector (GenScript, Nanjing, China). To exclude exon 17, the wild-type plasmid and a gBlock sequence (Integrated DNA Technologies, Coralville, IA, USA) lacking exon 17 were digested with 10U of *Acc65*I (Thermo Fisher Scientific, Waltham, MA, USA) and *Fse*I (New England Biolabs, Ipswich, MA, USA). The two fragments were ligated and transformed in GT115 competent cells (InvivoGen, San Diego, CA, USA) as described previously [31].

The expression and purification of WT and *ABCA4* variants were carried out as previously described [39]. Briefly, HEK293T cells were transfected with plasmid DNA (5 µg/10 cm plate) using 1 mg/ml polyethylenimine (PEI) MAX (Polysciences, Warrington, PA, USA) at a ratio PEI(µl):DNA(µg) 3:1. Cells were grown in a humidified incubator (5% CO_2_) at 37 °C in Dulbecco’s modified Eagle’s medium supplemented with 8% bovine growth serum, 200-µM l-glutamine and 1X antibiotic–antimycotic (Thermo Fisher Scientific, Waltham, MA, USA). After 24 h, cells were solubilized in buffer A (25 mM 4-[2-hydroxyethyl]-1-piperazineethanesulfonic acid, pH 7.4, 0.15 mg/ml brain polar lipid (BPL), and 0.07 mg/ml 1,2-dioleoyl-sn-glycero-3-phosphoethanolamine (DOPE), 150 mM NaCl, 1 mM MgCl_2_, 10% glycerol, and 1 mM dithiothreitol) containing 18 mM CHAPS detergent and protease inhibitor cocktail by slowly adding one plate of cells resuspended in 100 µl of buffer A to 0.5 ml solubilization buffer. The solution was stirred for 30 min at 4 °C and subsequently centrifuged for 10 min at 100,000*g* in a Beckman Optima TL centrifuge using a TLA110.4 rotor. The supernatants from cell lysates were mixed with 50 µl of Rim-3F4 Sepharose 2B immunoaffinity matrix for 1 h at 4 °C. The matrix was washed six times in buffer A containing 10 mM CHAPS. The ABCA4 protein was eluted two times with 60 µl each in the same buffer containing 0.5 mg/ml 3F4 peptide at 18 °C for 60 min.

An aliquot was analyzed on Coomassie blue-stained SDS gels for quantification of protein expression. The remaining sample was directly used for ATPase activity assays using the ADP–Glo Kinase Assay luminescence detection protocol (Promega, Madison, WI, USA). Briefly, a 20-µl ATPase reaction was performed by incubating 20–40 ng of protein with either 1 µl of 0.8 mM all-trans retinal (ATR) or buffer for 15 min followed by the addition of 4 µl of 2.5 mM ultrapure ATP for 40 min at 37 °C. Each reaction was done in triplicate. Six microliters of each reaction were then added to a well of a 384 Corning white polystyrene microtiter plate, followed by 6 µl of ADP–Glo reagent and 12 µl of kinase detection reagent. The luminescence signal was detected using on a Molecular Device SpectraMax M3 spectrometer equipped with SoftMax Pro 5.4 software. The ATP-deficient mutant ABCA4-MM in which the lysine residues in the Walker A motif of NBD1 and NBD2 were substituted for methionine, p.(Lys969Met), and p.(Lys1978Met), respectively, was used to subtract background luminescence [40].

For the localization study, HEK293T cells were grown on polylysine treated glass coverslips and transfected as described above. After 24 h, cells were fixed in 2% paraformaldehyde/0.lM phosphate buffer, pH 7.2 for 15 min and washed with 0.lM phosphate buffer 3 × 15 min.

Cell membranes were blocked and permeabilized with 10% normal goat serum, phosphate buffer and 0.1% Triton X-100 for 30 min. ABCA4 was detected using monoclonal RIM-3F4 and Alexa Fluor 568 anti-mouse secondary antibody (Thermo Fisher Scientific, Waltham, MA, USA) and Calnexin using polyclonal anti-calnexin (Abcam, Cambridge, UK) and Alexa Fluor 488 anti-rabbit secondary antibody (Thermo Fisher Scientific, Waltham, MA, USA) in phosphate buffer containing 0.3% normal goat serum and 0.075% Triton X-100. Fluorescence was detected using a Zeiss LSM700 confocal microscope (Carl Zeiss, Oberkochen, Germany).

ARPE-19 (ATCC, Manassas, VA, USA) cells were cultured in Advanced DMEM/F-12 medium (Thermo Fisher Scientific, Waltham, MA, USA) with 10% Fetal Bovine Serum (Biowest, Nuaillé, France) at 37 °C with 5% CO_2_. At 80–90% confluency, the cells were harvested and 3 × 10^4^ cells were seeded in a µ-Slide 8 Well chambered coverslip (ibidi GmbH, Gräfelfing, Germany). The cells were transfected 24 h later with 250 ng of mutant or wild-type plasmid and incubated at 37 °C for additional 48 h. Part of the cells were used for immunoblotting for quantification of protein expression by following the previously described protocol [31], and part was used for immunostaining. For the latter, the cell cultures were rinsed with ice-cold PBS (Thermo Fisher Scientific, Waltham, MA, USA) with 5% Blocker BSA (Thermo Fisher Scientific, Waltham, MA, USA), fixed in cold 4% paraformaldehyde (Thermo Fisher Scientific, Waltham, MA, USA) and incubated at RT for 20 min. Next, these were permeabilized with ice-cold PBS + 0.1% Triton X-100 (Promega, Madison, WI, USA) for 10 min and blocked with PBS + 5% BSA for 1 h at 4 °C. The cells were incubated with anti-Calnexin antibody (1:200, Thermo Fisher Scientific, Waltham, MA, USA) and anti-ABCA4 Antibody, clone 3F4 (1:100, Abcam, Cambridge, UK) overnight at 4 °C. The nuclei were stained with Hoechst 33342 (1:1000, Thermo Fisher Scientific, Waltham, MA, USA). The images were collected with an LSM 800 confocal microscope (Carl Zeiss, Oberkochen, Germany) using a 20 × objective and analyzed with ZEN Blue edition (Carl Zeiss, Oberkochen, Germany).

### Antisense oligonucleotide design and screening in wild-type WERI-Rb-1 cells

The AONs were designed as described previously by Kaltak et al. [31] as an oligo-walk in order to target the *ABCA4* exon 17 and the adjacent 60 nt and 16 nt of introns 16 and 17, respectively.

WERI-Rb-1 cells were grown as grape-like clusters in suspension in culture flasks with Roswell Park Memorial Institute 1640 Media (RPMI 1640, Thermo Fisher Scientific, Waltham, MA, USA) enriched with 10% Fetal Bovine Serum (FBS, Biowest, Nuaillé, France) at 37 °C with 5% CO_2_.

5 × 10^5^ cells were transferred in 12-well plates and the AONs (ProQR Therapeutics, Leiden, the Netherlands), reported in Additional file 1: Table S1, were added to the cell culture medium at 10 µM as described previously [26]. The RNA was extracted, reverse-transcribed into cDNA and the isoform content was investigated using digital PCR (dPCR) by following the previously reported protocol [31]. The measurement of total *ABCA4* was conducted in the latter by employing an assay consisting of primers that bound to exons 8 and 9, along with a probe targeting the junction of these two exons. Similarly, *ABCA4* Δexon 17 was assessed using an assay that comprised primers binding in exons 16 and 18, and a probe targeting the junction of these two exons. The sequences of the assays are reported in Additional file 1: Table S2.

### Generation of wild-type retinal organoids

StemRNA Human induced pluripotent stem cell line (iPSC) 771-3G (ReproCell, Yokohama, Japan) was cultured in 6-well plates coated with Corning^®^ Matrigel^®^ hESC-Qualified Matrix (Corning, NY, USA) containing 2 ml of mTeSR™1 medium (Stemcell Technologies, Vancouver, Canada) enriched with 25% mTeSR™1 5X Supplement (Stemcell Technologies, Vancouver, Canada) and 1% Penicillin/streptomycin (Sigma-Aldrich, Burlington, MA, USA). The iPSCs were differentiated in ROs by applying the protocol described by Hallam et al. [41].

### Antisense oligonucleotide treatment in wild-type retinal organoids

AONs were administered to 190-days-old ROs at a 10 µM concentration; the culture medium was fully removed and fresh medium with AON was added. Every two days half of the culture medium was removed and replaced by fresh culture medium; this “wash-out” regimen was followed for 4 weeks. Next, the culture medium was removed completely, the ROs were washed in PBS and harvested in 300 µl of TRIreagent (Zymo Research, Irvine, CA, USA). The samples were snap frozen in liquid nitrogen and stored at − 80 °C. The ROs were lysed with a 25-gauge needle, and the RNA was extracted with the Direct-Zol RNA MicroPrep kit (Zymo Research, Irvine, CA, USA) according to the manufacturer’s instructions. 100 ng of extracted RNA was reverse transcribed in cDNA as described above. The samples were analyzed with isoform-specific assays for total *ABCA4* and *ABCA4* Δexon 17 (Additional file 1: Table S2) as described above.

To ensure the quality of ROs, the levels of retinal markers *CRX*, *OPN1MW*, and *RHO* were utilized, with a minimum threshold of more than 1000 copies/ng RNA. Samples that fell below all three thresholds were not included in the analysis. The positive and negative partitions were distinguished based on manually set thresholds in all experiments. To adjust for variations in cDNA input, the identified *ABCA4* isoforms were normalized to the geometric mean of *CRX*, *RHO*, and *OPN1MW*.

The expression of additional retinal markers, i.e. *NR2E3* (Hs00183915_m1, Thermo Fisher Scientific, Waltham, MA, USA) and *NRL* (Hs00172997_m1, Thermo Fisher Scientific, Waltham, MA, USA), was assessed to assure the integrity of marker expression before and after AON treatment.

### Transcript data analysis

Using the QIAcuity Software Suite (Qiagen, Hilden, Germany), the positive and negative populations were separated with manually set thresholds, and the positive population was considered for further calculations. The formula used to calculate the percentage of AON-induced *ABCA4* exon 17 skip in WERI-Rb-1 cells and ROs was the following: % *ABCA4* Δexon 17 relative to total *ABCA4* = *ABCA4* Δexon 17/*ABCA4* exons 8-9.

### In silico off-target assessment for the best performing antisense oligonucleotides

To determine the potential off-target effects of AONs used in the treatment administered to ROs, an in silico analysis on human genomic DNA and transcriptome described previously was performed. *Homo sapiens* DNA (RefSeq_Gene) and mRNA (refseq_rna) sequence databases were investigated manually with the Basic Local Alignment Search Tool (https://blast.ncbi.nlm.nih.gov, accension date 15 May 2023).

### Statistical analysis

All graphs represent data as mean ± SEM. The mean percentages of the AON efficiency and expression of retinal markers were analyzed for statistical significance vs. untreated or scrambled AON using GraphPad Prism 9 with ordinary one-way ANOVA test followed by Dunnett’s multiple comparison test. The mean numbers of CSS variants nearby multiple-of-three exons and non multiple-of-three-exons were investigated for statistical significance with an unpaired two-tailed t test with Welch’s correction. P ≤ 0.05 was considered statistically significant.

## Results

### Ophthalmogenetic findings in a STGD1 proband carrying *ABCA4* c.2653+1G>A and a null allele

**Fig. 1:**
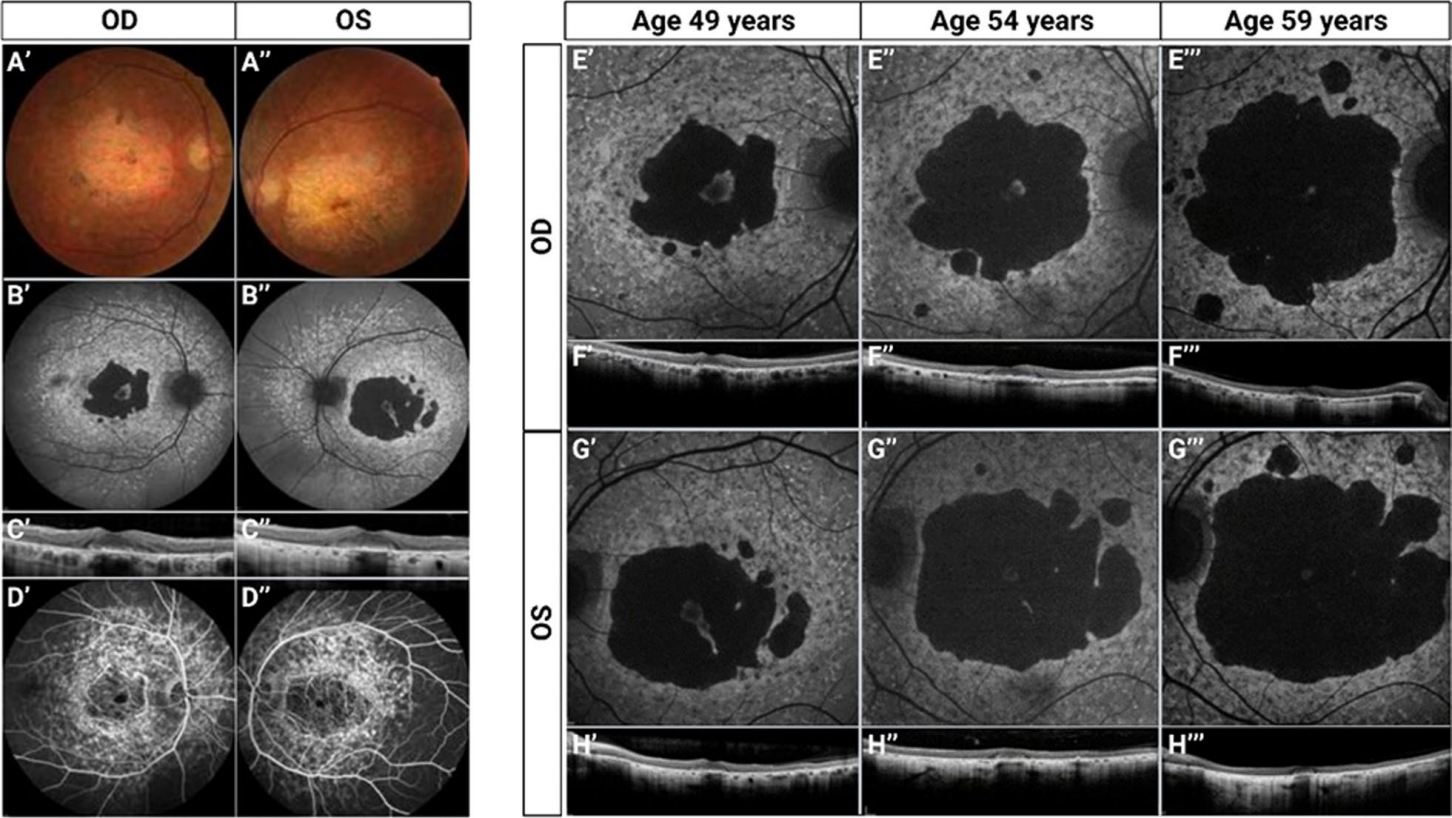
Multimodal imaging of both eyes of the proband. **A**–**D** Analysis at baseline performed at proband’s age of 49 years. (OD, oculus dexter or right eye; OS, oculus sinister or left eye) (**A′**, **A″**) Color fundus photographs reveal extensive macular atrophy with some pigment clumps more noticeable on the right eye along the border of the atrophy. There is some retro foveal retinal tissue remaining. **B′**, **B″** Fundus autofluorescence (FAF) images where a large amount of hyperautofluorescent coalescent flecks is evident extending outside the vascular arcades but remaining within the posterior pole. The foveal sparing is greater on the right eye. Peripapillary sparing is present in both eyes. **C′**, **C″** Spectral domain optical coherence tomography (SD-OCT) scan images passing through the fovea, shows clumping of the retinal pigment epithelium and extensive atrophy of the external retinal layers with retro foveal sparing. **D′**, **D″** Fluorescein angiography mid phase images of the posterior pole. A window defect is present on both eyes in the areas presenting atrophy and beyond the posterior pole choroidal silence is present. This is not noticeable on the posterior pole due to the large amount of coalescent flecks. **E**, **G** FAF images of the macular region dating from presentation at proband’s age of 49, 54 and 59 years. The progression of the atrophy is evident. **F**, **H** SD-OCT scans passing through the fovea show loss of retinal tissue corresponding to the progression of central atrophy

We identified a French STGD1 proband that carried a previously reported null variant c.735T>G, p.(Tyr245*) [42] and a novel canonical splice site variant c.2653+1G>A. Segregation analysis in the mother and brother revealed that these two variants were in *trans*. Interestingly, this proband presented with late onset symptoms (at 43 years of age), but consulted a ophthalmologist just 6 years later, in 2010, when fundus abnormalities were detected. The initial best corrected visual acuity (BCVA) measured in Monoyer scale was 10/10 for both eyes (Snellen equivalent 20/20). At the time, the main reported complaints were hemeralopia and photophobia. The initial exams revealed normal anterior segments on both eyes at slit lamp examination and no major refractive issues. Fundus examination revealed the appearance of a flecked retina with obvious signs of atrophy, as shown in Fig. 1A. Fundus autofluorescence (FAF) identified hyperautofluorescent lesions localized at the macula and extending about one disc diameter outside the vascular limits of the macula (Fig. 1B). Extensive central retinal atrophy was also noted. The presence of foveal sparing was confirmed by spectral domain optical coherence tomography (SD-OCT) (Fig. 1C). FAF also revealed peripapillary sparing. Fluorescein angiography (FA) showed signs of choroidal silence in the very early phases, more evident in the fleck and atrophy free zones extending beyond the mid periphery (Fig. 1D). Full field electroretinography (ffERG) showed subnormal scotopic and photopic responses for both eyes compared to the normative data for our center (Additional file 1: Fig. S1). BCVA remained at 20/20 for five years, then declined progressively and symmetrically over the next few years diminishing to 20/25 on 2016. In 2019, BCVA further decreased to 20/50 on both eyes and has been stable since. This is secondary to the foveal sparing seen up until his last exam on SD-OCT (Fig. 1E–P).

Given the presence of combinations of severe and mild *ABCA4* variants in other cases with late-onset STGD1, we hypothesized that variant c.2653+1G>A results in significant remaining activity of the ABCA4 protein.

## c.2653+1G>A results in exon 17 skipping in a midigene splice assay

**Fig. 2:**
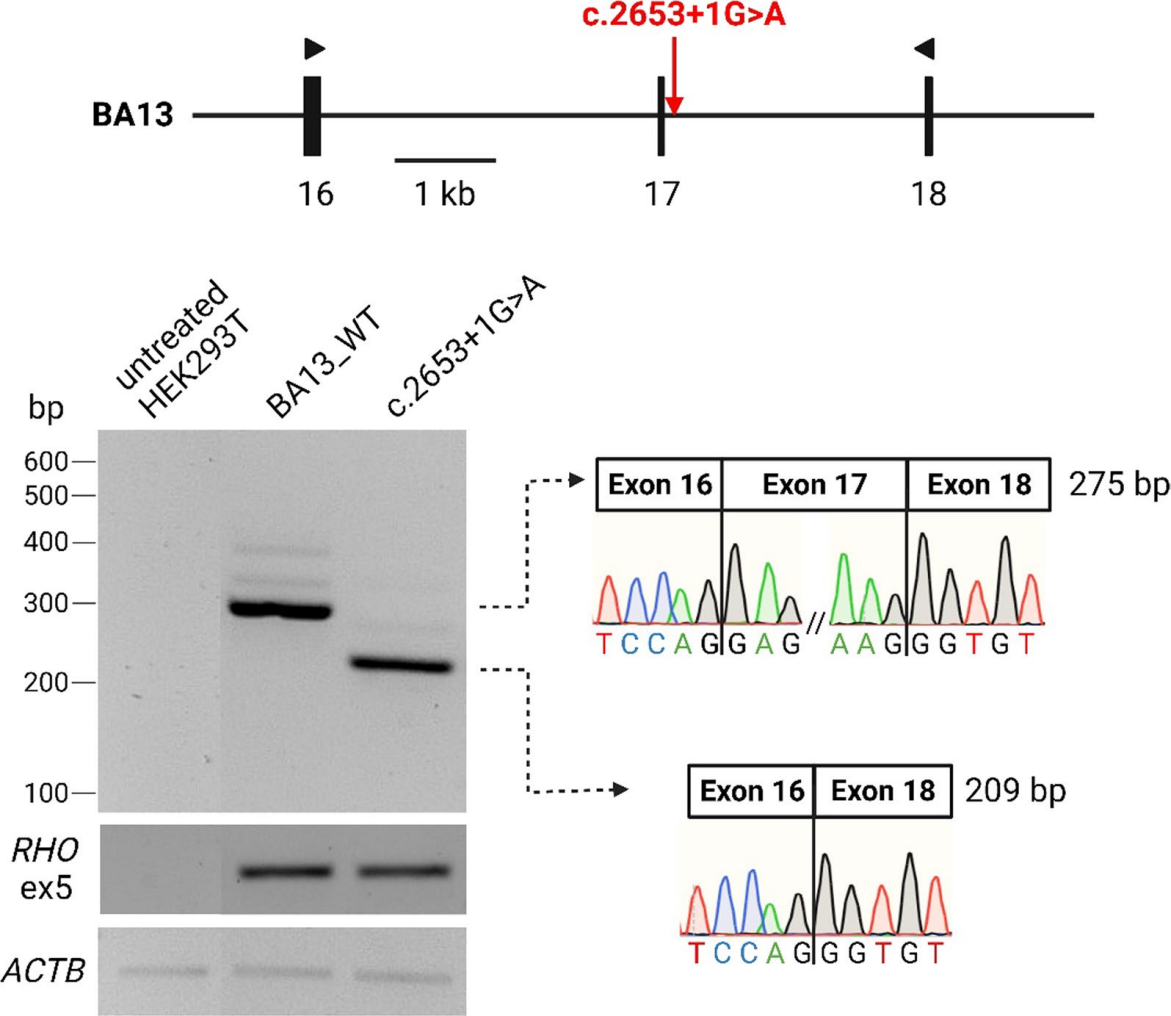
Variant c.2653+1G>A results in complete skipping of exon 17. The *ABCA4* genomic sequence flanked by *rhodopsin* exons 3 and 5. cDNA products were amplified using primers situated in exons 16 and 18, represented with triangles. The mutant construct shows complete exon 17 skipping as shown by Sanger sequencing

SpliceAI [43] predicted that c.2653+1G>A weakens the exon 17 splice acceptor site (SAS; delta score 0.63) and splice donor site (SDS; delta score 0.98), and very likely leads to complete skipping of exon 17. To confirm this predicted effect, we included c.2653+1G>A in the previously described BA13 wild-type (WT) midigene [37] that contains *ABCA4* exons 16 through 18, and flanking intronic sequences (8258 bp; g.94521462–g.94513205 [GRCh37)]. Although the variant was introduced using a high-fidelity DNA polymerase, the analysis of the entire mutant plasmid sequence identified a single PCR-introduced polymorphism that is unlikely to impact splicing (Additional file 2: Table S3). The rest of the sequence was consistent with previously reported analyses [36]. The expression of constructs was carried out in HEK293T cells that do not express endogenous *ABCA4*, and the reverse transcription (RT)-PCR of the cDNA confirmed the exon-skipping effect of the variant which was not observed for the WT construct (Fig. 2). As this is a full effect, the protein product is p.Asp864_Gly885del (referred to as ΔAsp864_Gly885 hereafter).

### ΔAsp864_Gly885 shows partial ABCA4 ATPase activity and localizes to the same cellular structures as wild-type ABCA4

**Fig. 3:**
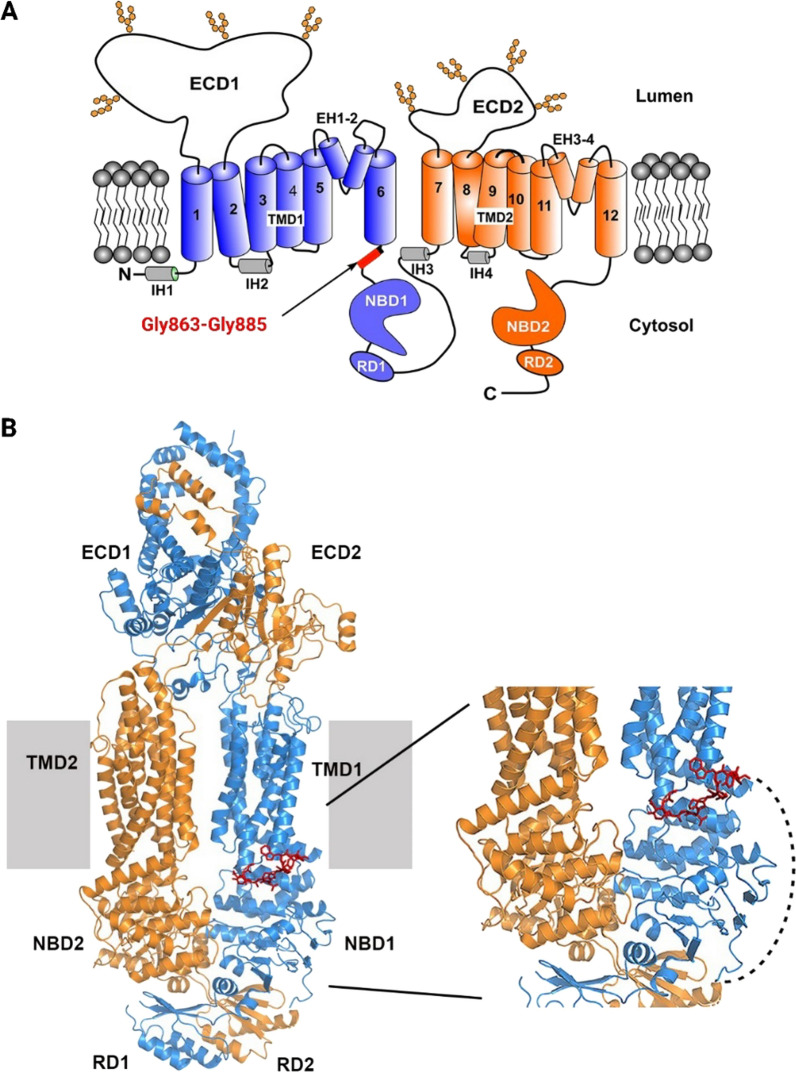
Topological and molecular structure of ABCA4. **A** Topological diagram of ABCA4 showing the various domains of ABCA4 and the location of the peptide segment encoded by exon 17. ECD, exocytoplasmic domain; NBD, nucleotide-binding domain; TMD, transmembrane domain; RD, regulatory domain. Hexagons denote oligosaccharide chains extending from the ECDs. EH, external helix; IH, internal helix. The 22 amino acid-segment encoded by exon 17 is depicted as a red box; **B** Molecular structure of ABCA4 (PDB: 7LKP). Red sticks show the part of the 22 amino acid-segment encoded by exon 17 that could be modeled (Gly-Asp-Tyr-Gly-Thr-Pro-Leu-Pro-Trp-Tyr-Phe). Right: Enlarged region of ABCA4 containing the segment encoded by exon 17. Dashed line represents the remaining peptide encoded by exons 17 and 18. This segment could not be modeled in any of the ABCA4 structures, presumably due to the flexible nature of this segment of the protein. N-half and C-half are colored blue and orange, respectively

ABCA4 contains a 52 amino acid linker segment (Gly863-His914) encoded by exons 17 and 18 that extends from transmembrane 6 of TMD1 to the start of the NBD1 (Fig. 3A). Within exon 17, residues Gly863-Phe873 could be modeled in the ABCA4 structure in its unbound state (Fig. 3B). In contrast, the remaining linker segment (Leu874-His914) encoded by the remaining part of exon 17 and all of exon 18 was not observed in any of the structures of ABCA4, most likely due to the highly flexible nature of this segment.

**Fig. 4:**
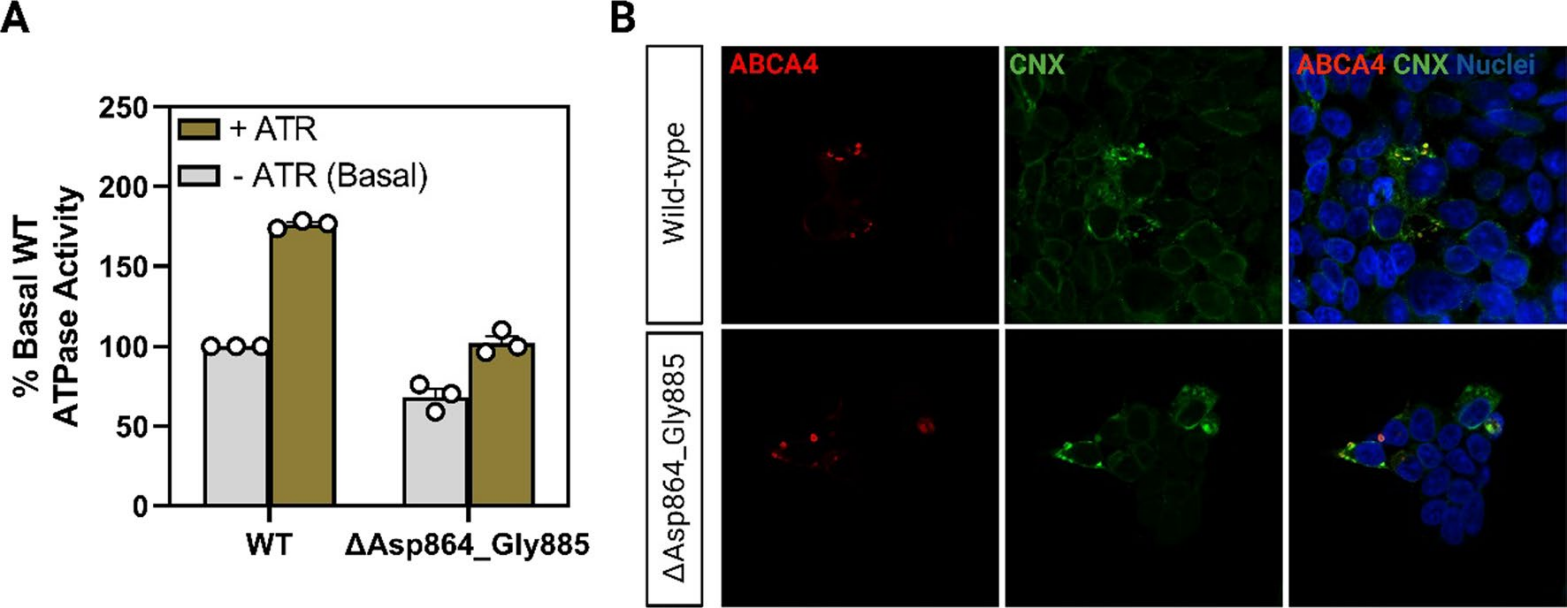
ABCA4 ΔAsp864_Gly885 shows significant activity and displays a subcellular localization in HEK293T cells similar to ABCA4 WT**. A** ATPase activity of wild-type ABCA4 (WT) and the ΔAsp864_Gly885 variants in the presence and absence of all-trans retinal (ATR) expressed as the percentage activity in the absence of ATR (basal activity). The data is shown as mean ± SEM, n = 3 for each construct and represents 3 independent experiments. **B** HEK293T cells were transfected with the ABCA4 plasmids, labeled for ABCA4 (red) and calnexin as an ER marker (green) and counterstained with DAPI nuclear stain (blue). The ΔAsp864_Gly885 variant is associated with intracellular vesicles, that resemble vesicles observed for WT ABCA4. A fraction of the truncated protein is retained in the ER

To assess the function of ABCA4 lacking the 22 amino acids encoded by exon 17 variants, ATPase activity of the ΔAsp864_Gly885 protein variant was compared to the activity of wild-type (WT) ABCA4. For these studies, the WT ABCA4 and the ΔAsp864_Gly885 variant were expressed in HEK293T cells and purified by immunoaffinity chromatography. The ΔAsp864-Gly884 variant expressed at similar levels to WT ABCA4 as analyzed on Coomassie blue-stained gels (Additional file 1: Fig. S2) and displayed all-trans retinal (ATR) activated ATPase activity (Fig. 4A). A 1.8 fold increase in ATPase activity in the presence of ATR was observed for WT ABCA4 in agreement with previous studies [39], while the ΔAsp864_Gly884 variant showed a smaller 1.5 fold increase in activity (Fig. 4A). The level of ATR-stimulated activity of the variant was 58.0 ± 4.2% of the activity measured for WT ABCA4. These studies indicate that ABCA4 lacking the peptide segment encoded by exon 17 retains considerable functional activity, a property consistent with a predicted moderate (intermediate) STGD1 phenotype for an individual with a *trans* null allele based on functional studies [39, 44].

Additionally, the localization of ΔAsp864_Gly885 and of the WT protein was investigated in HEK293T (Fig. 4B) and ARPE-19 cells (Additional file 1: Fig. S2E). Immunocytochemistry analysis revealed that ΔAsp864_Gly885 trafficked to subcellular vesicles, which resembled the localization of the WT protein in both cell types. However, a fraction of the truncated protein co-localized with the endoplasmic reticulum (ER) that was stained with the antibody against calnexin (CNX), as opposed to the WT protein which was not retained in the ER. These results point to a partial misfolding of ΔAsp864_Gly885.

### In silico analysis of other in-frame skipped exons in *ABCA4*

To determine the severity of transcripts lacking in-frame exons other than exon 17 in *ABCA4*, we performed in silico analyses of canonical SAS and SDS variants in the other 16 exons consisting of a multiple of three base pairs. The effect of these variants on splicing was predicted by SpliceAI and listed in Additional file 3: Table S4. A clear prediction for complete exon skipping was found for all theoretical canonical splice site (CSS) variants located at 11 out of 34 exon–intron junctions in the exons 7, 11, 17, 18, 20, 32, 34 and 49. To assign a severity label to the implicated CSS variants, we consulted published data of previously screened probands for their age of onset and the nature of the *ABCA4* variant in *trans*. We implemented two rules to categorize the CSS variants as severe: 1. the variant in *trans* was previously classified as mild, and therefore, according to a generally accepted genotype–phenotype model, a second severe allele has to be present in order for STGD1 to occur, or 2. the age of onset was before the age of ten, which generally is found when there are two severe *ABCA4* alleles.

According to the analysis in Additional file 4: Table S5, the majority of investigated CSS variants are presumed to lead to *ABCA4* transcripts that contain a premature stop codon or frameshift at locations that point to severe effects for these CSS variants. For one reported variant found in homozygosity (c.1554+1G>C) we were unable to find the age of onset, however, the severe cone-rod dystrophy diagnosis allowed to assume the variant’s severe effect. In addition, the amino acid sequences encoded by the 17 in-frame exons were located within the ABCA4 domains to predict their potential role in the structure and function of the protein. Specifically, exons 6, 7, 10, 11, 13, 29, 32 and 34 translate into parts of the ECD1 and ECD2, forming highly interlaced α-helices, glycosylation sites at positions N415, N444 and N504, and a recognition site for N-ret-PE mediated by Arg587 [12, 13]. Other in-frame exons (exons 20, 22, 42, 45 and 47) contribute to α-helical-, β-structures and highly conserved Walker A and B motifs in the NBDs which are characteristic for all proteins that are sustained by ATP-hydrolysis. Exons 15 and 49 encode parts of TMD1 and RD2, respectively (Additional file 5: Table S6). These domains contribute to the correct folding of the protein, as discovered in previous studies [45]. Together with exon 17, exon 18 is predicted to encode the linker that connects TDM1 with NDB1. Unlike other in-frame exons, the amino acids encoded by exons 17 and 18 do not display an available structure, which suggests the presence of a highly flexible region that could potentially endure modifications without causing major disruptions within the structure and function of the protein.

Altogether, this data points to the presumption that in-frame skipping of any of these exons, with the exception of exon 17, is predicted to result in severe or deleterious alleles.

When compared to all *ABCA4* CSS variants, we found no statistical differences in frequencies of CSS variants located nearby exons with a multiple of three nucleotides and those located nearby non multiple-of-three nucleotide exons (p = 0.35). The list of all CSS *ABCA4* variants can be found in Additional file 6: Table S7.

### AON treatment induces the generation of *ABCA4* Δexon 17 RNA at significant levels

**Fig. 5:**
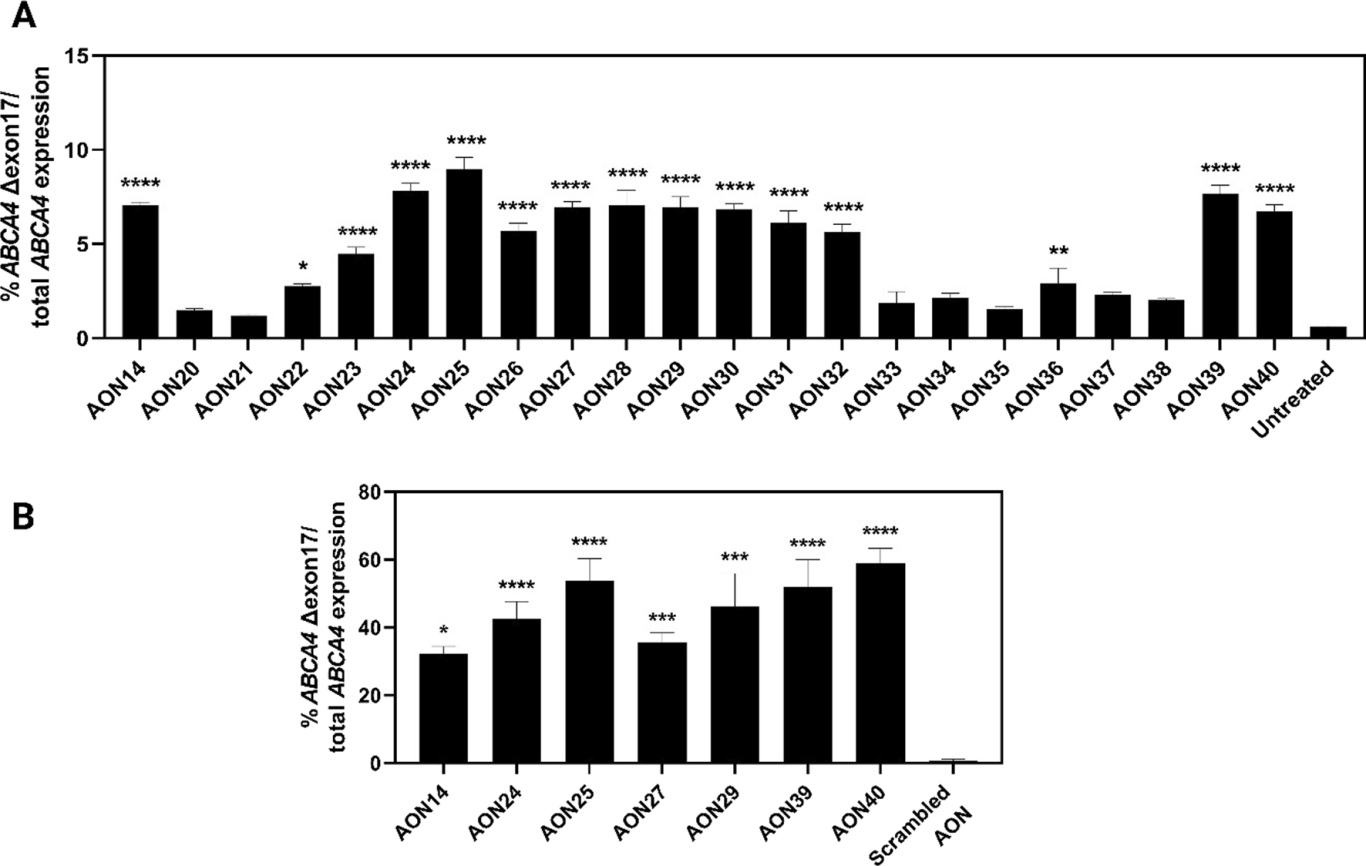
AON-based treatment achieved significant levels of *ABCA4* Δexon 17 transcripts. **A** The percentage of *ABCA4* mRNA lacking exon 17 in WT WERI-Rb-1 cells after 48 h long AON treatments, n = 3. **B** The percentage of *ABCA4* mRNA lacking exon 17 in WT retinal organoids is significantly increased upon exposure to the most effective AONs in a 4-week long wash-out treatment, n = 6. The data is shown as mean ± SEM. Asterisks display the significant differences vs. untreated or scrambled AON (*p ≤ 0.05, **p ≤ 0.01, ***p ≤ 0.001, ****p ≤ 0.0001)

The LOVD database (https://www.lovd.nl/ABCA4; accessed on 20th January 2023) contains 14 disease-associated variants that reside in exon 17 (Additional file 7: Table S8). Thirteen of these are considered to have a severe effect, as classified previously by Cornelis et al. [17]. Furthermore, most of these variants were classified as “Pathogenic” or “Likely pathogenic” based on ACMG criteria, one is considered “Variant of unknown significance” [c.2617T>A, p.(Phe873Ile)] and one variant could not be categorized [c.2619T>A, p.(Phe873Leu)]. As ABCA4 lacking Asp864_Gly885 shows significant ATPase activity and is partially located in the same cellular structures in HEK293T and ARPE-19 cells, STGD1 patients carrying the above-mentioned variants are likely to benefit from an AON-based treatment that enforces complete skip of exon 17. Thus, an oligo-walk was designed that consisted of 40 AONs that target the entire exon 17 and adjacent introns, and set out to identify the best candidates able to induce the ‘in-frame’ skip. The first screening was carried out in WT WERI-Rb-1 cells with 19 different AONs consisting of 20–22 nt. The molecules were delivered at a 10 µM concentration by gymnosis. AON14 showed the highest efficiency, albeit modest, by leading to 6.4 ± 0.2% of *ABCA4* Δexon 17 mRNA and its binding region was defined as the target region for the second AON screening (Additional file 1: Fig. S3). This consisted of a set of 22 AONs of 17–19 nt as previously it was shown that shorter AONs achieved a higher desired effect over longer AONs, likely due to a favored cellular uptake [31]. As shown in Fig. 5A, digital PCR detected 9.0 ± 0.7% of *ABCA4* Δexon 17 mRNA isoforms, as opposed to the untreated group where this isoform was present at 0.6 ± 0.1%. As expected, the best AON candidates targeted strong splice enhancer motifs either within the sequence of exon 17 or nearby the downstream splice site, which caused exclusion of the exon (Additional file 1: Fig. S4). Next, these AONs were administered at the same concentration to 190-days-old WT retinal organoids (ROs) and the treatment consisted of a wash-out regimen implemented for 28 days. *ABCA4* Δexon 17 mRNA was quantified at significantly higher levels in a range between 32.2 ± 2.1% and 58.8 ± 4.6% post-treatment; the control group treated with a scrambled AON contained no truncated transcript (Fig. 5B). In all groups, the expression of retinal markers (*ABCA4, CRX, NRL, NR2E3, and RHO*) displayed a consistent pattern, except for *OP1NMW* that expressed at significantly different levels in ROs treated with AON24 and AON25 when compared to the scrambled AON group (Additional file 1: Fig. S5).

### In silico analysis of the off-target effects of the best performing antisense oligonucleotides

The seven AONs used in the treatment of ROs were analyzed for their possible off-target effects in the human genome and transcriptome. As reported in Additional file 8: Table S9, none of the AONs showed full complementarity against the human DNA or mRNA, except for the intended target in *ABCA4*. Some genomic targets were identified when allowing for one or two mismatches with all AONs, except for AON14 where this was not observed. As the binding location in the genomic targets were either in the intragenic regions or in deep intronic regions ≥ 577 bp distant from the adjacent exon junctions, the AONs are not likely to interfere with the splicing of these genes. However, an exception was identified with AON25, that showed a binding location with 2 mismatches 47 nt upstream the first exon in *DNAJC5.* In addition, AON27 and AON40 showed a two-mismatch complementarity with the mRNA of *RUNDC3A* and *GALNT5*. These genes do not express protein in the retina and therefore are unlikely to be relevant regarding off-target effects of AONs.

## Discussion

The main cause underlying STGD1 is reduced or absent phospholipid-retinoid transport exhibited by the ABCA4 transporter, which so far remains without a treatment. Several pre-clinical studies reported promising advances with novel therapies that apply different means to tackle the genetics of the disease, either by reverting a variant’s disease-associated effect on the genomic level with CRISPR-Cas9 or on the level of transcripts by implementing RNA modulating therapies (AONs) [30–35, 46]. Despite being too large for introduction in AAV vectors, ABCA4 was successfully delivered and recombined in mouse and pig retinas by combining two AAV vectors and split inteins [47]. All mentioned research is focused on restoring the dysfunction of the diseased protein to its wild-type settings. In this study, we report skipping of the in-frame exon 17 in *ABCA4* due to a CSS variant, as observed in a STGD1 proband, that leads to a shortened protein that retained significant levels of ATR-activated basal activity when compared to wild-type ABCA4. This discovery led to the development of potent AONs able to induce skipping of exon 17, which could potentially delay or stop the STGD1 phenotype progression caused by protein-truncating and severe missense variants in *ABCA4* exon 17. This novel therapeutic approach, for the first time, implements skipping of a coding region in *ABCA4*, and widens the arsenal of therapeutic possibilities focused to alleviate the progression of clinical features associated with STGD1.

The *ABCA4* gene encompasses 50 exons that is translated into a 2273 amino acid polypeptide. Seventeen of these exons are composed of multiples of three nucleotides. Theoretically, their absence in the mRNA would leave the open reading frame intact, leading to proteins that lack between 11 and 74 amino acids. However, the majority of ‘in-frame’ coding sequences in *ABCA4* encode crucial protein domains that are considered essential for the protein’s function. In silico analysis of previously reported CSS variants, predicted to lead to complete skip of numerous in-frame exons, points to their pathogenic nature when taking into consideration the reported allele in *trans* and onset of age in STGD1 probands (Additional file 1: Table S5). Altogether, the deletion of the structural elements encoded by all other in-frame exons in *ABCA4* would, most likely, severely impair the protein’s action, as they play important roles in protein stability, folding, interaction and transport of substrates, and ATP-hydrolysis. Exons 17 and 18 however encode parts of the polypeptide that localize entirely in the linker between TMD1 and NBD1. This region lacks a well-defined structure, and it indicates a highly flexible portion of the protein, which might only be partially essential for the protein’s expression and function. Even though the presence of this seemingly simple element appears to be less substantial when compared to other protein domains, there is little knowledge on its exact function. Of note, a deletion of exon 18 was identified in two individuals [48]. Interestingly, the detected alleles in *trans* in both cases carried the c.2588G>C p.[Gly863Ala,Gly863del] variant. As c.5603A>T in 2003 was not yet appreciated as a hypomorphic but causal variant, it probably was present in *cis* with c.2588G>C, and together these two variants form a mild but fully penetrant allele. This finding strongly suggests that skipping of the in-frame exon 18 has a severe effect.

We identified a novel CSS variant downstream exon 17, c.2653+1G>A, in a French STGD1 proband that carried the prior characterized c.735T>G, p.(Tyr245*) variant in *trans*. Since this case displayed late-onset STGD1, it allowed to hypothesize that c.2653+1G>A leads to translation of ABCA4 with significant remaining functionality. We confirmed the variant’s exon-skipping nature on exon 17 by exploring its expression in midigene-transfected HEK293T cells. The mild nature of the protein lacking the 22 amino acids (ΔAsp864_Gly885) encoded by exon 17 was determined by a functional assay, where the retained ATR-activity was measured at 58% when compared to the WT ABCA4. Furthermore, plasmid-induced overexpression of ABCA4 in HEK293T and ARPE-19 cells showed that ΔAsp864_Gly885 localized in vesicle-like structures previously attributed to the WT ABCA4 [49, 50]. These results suggest that exon 17 is the only in-frame exon that can be omitted from the *ABCA4* coding sequence without having a deleterious effect. In addition, the linker structure expressed by this exon most likely has a moderate role in the spectrum of functions exhibited by ABCA4. The significance of this linker for the function of ABCA4 is not known, but it is possible that it serves as a flexible segment that allows ABCA4 to undergo the large conformational change observed upon the binding of ATP to the NBDs during the substrate transport cycle. However, the exact functional role requires further studies.

The data presented above allowed to develop an AON-based treatment able to induce exon 17 skipping in *ABCA4*. In order to define the patient group that would potentially benefit from this type of therapy, we identified 14 previously reported *ABCA4* variants that reside in exon 17. Most of these variants have a deleterious effect on the protein, with the exception of c.2608C>T, p.(Pro870Ser), c.2617T>A, p.(Phe873Ile), c.2617T>C, p.(Phe873Leu) and c.2619T>A, p.(Phe873Leu), for which this is as yet unknown. Nearly all variants in exon 17 are considered “Severe” according to Cornelis et al. [17] and are labelled as “Likely pathogenic” and “Pathogenic” using the ACMG categorization. Thus, STGD1 probands carrying a severe variant in exon 17 would benefit from the AON-mediated exon skip treatment. AONs have emerged as promising therapeutic intervention for treatment of several inherited retinal diseases; two programs continued the investigations in clinical trials exploring the potency of AONs in rescuing the severe phenotypes of Leber congenital amaurosis 10 (LCA10) and *USH2A*-associated Usher syndrome and retinitis pigmentosa (RP), by applying sepofarsen and ultevursen, respectively. The first clinical trial for sepofarsen showed promising results regarding its safety profile and mechanism of action and identified five out of 11 participants that displayed clinically significant improvements in best corrected visual acuity, full-field stimulus test and mobility course [51]. While sepofarsen removes a pseudoexon, whose insertion is caused by the recurrent c.2991+1655A>G variant, to restore the wild-type splicing in *CEP290*, ultevursen implements skipping of the in-frame exon 13 in *USH2A*, which shortens the protein while retaining its residual activity. *USH2A*-coded usherin contains ten repetitive epidermal growth factor (EGF)-like domains that allow for protein shortening. The AON-mediated ‘in-frame’ exon 13 skipping, which contains frequent and severe *USH2A* variants, led to a well-tolerated fusion of EGF-like domains 4 and 8; moreover, the results report a properly localized protein with clinically relevant residual activity [26]. As opposed to usherin, ABCA4 lacks repetitive elements whose absence would translate into a protein with residual activity. However, we identified that the lack of Asp864_Gly885 encoded by exon 17 results in a shortened linker between TMD1 and NBD1. Although this approach results in a reduction of ABCA4 activity, the residual activity is still within acceptable levels for use as a therapeutic intervention. Additionally, the AONs described in this study exhibit physicochemical characteristics that are comparable to sepofarsen and ultevursen, which further strengthens their potential for future clinical applications targeting STGD1. However, further investigations into their pharmacological properties are necessary before proceeding with clinical studies.

The results across two AON screenings in a simpler cell model identified candidates that were employed in further screening in a more complex cellular model, 3D human ROs. We observed that the AONs induced higher levels of *ABCA4* Δexon 17 transcripts in ROs, as opposed to the effect observed in WERI-Rb-1 cells. This could be justified by the presence of AON-targeted splicing motifs that are most likely acting in a retina-specific manner when contained in a differentiated retinal environment. Previous studies have found that disease-associated isoforms specific to the retinal tissue exhibit differential expression in ROs or photoreceptor precursor cells (PPCs), compared to simpler cell cultures. In fact, the most frequent Leber congenital amaurosis-associated *CEP290* variant c.2991 + 1655A > G led to more prominent pseudoexon insertion in patient-derived ROs compared to patient fibroblasts [52]. This trend was also observed for two neighboring *ABCA4* intron 30 variants, which created or strengthened exonic splice enhancer motifs, thereby leading to the 345-nt pseudoexon insertion in patient-derived PPCs, but not in patient-derived fibroblasts [53]. Even though WERI-Rb-1 cells represent a common model to investigate AON-mediated suppression of gene expression and exon skipping due to endogenous expression of several IRD-associated genes and retina-like splicing mechanism [26, 54–56], ROs are considered to more closely resemble native retinal tissue. Their self-development can be compared to the natural development of the retina [57–59], for which reason they became a widely used in vitro model for investigation of disease development and applicable therapeutic compounds [32, 60–63]. Previously, the *ABCA4*-targeted AON treatment led to a highly restorative effect on the transcriptional and protein level in both gene-edited and patient-derived ROs. In addition, longer exposition to AONs increased their effect up to twofold when prolonging the treatment from 4 to 8 weeks (31). The extended effect over longer periods can be explained by the inactive portion of oligos entrapped by endosomes and their release over time.

## Conclusion

In conclusion, we provide the first evidence of successful exon skipping in *ABCA4*, resulting in a protein with significant levels of remaining functionality and a correct subcellular localization. Although the coding region of *ABCA4* captures multiple in-frame exons whose omission would, in theory, lead to a truncated but functional version of the protein, the high structural organization of ABCA4 presents a challenge for any exon-skipping therapeutic intervention.

While the impact of skipping other ‘in-frame’ exons on the residual activity of ABCA4 is yet unknown, we propose that the remaining ABCA4 activity upon skipping of exon 17 is an exception rather than a rule for any of the other ‘multiple of three nt’ exons. Here, we have shown an innovative AON-mediated therapeutic strategy that can induce skip of the entire exon 17 in *ABCA4* and could, potentially, offer therapeutic benefits to STGD1 patients that harbor severe variants in *ABCA4* exon 17.

### Supplementary Information

**Additional file 1:** **Figure S1. **Proband’s baseline full field electroretinography (ffERG) results (ISCEV protocol; MetroVision, Perenchies, France). **A** Moderately subnormal scotopic rod (dark adapted) responses for both left (OS, oculus sinister) and right eyes (OD, oculus dexter). **B** Moderately subnormal mixed response (dark adapted), of which the right eye shows a more diminished response. **C** Subnormal oscillatory responses (dark adapted) for both eyes (OP, oscillatory potential). **D** Very diminished cone responses (light adapted) in both eyes. **E** Subnormal flicker response (light adapted) for both eyes. **Figure S2. **Expression of *ABCA4 *constructs in HEK293T (**A**, **C**) and ARPE-19 (**B**, **D**, **E**) cells. **A** Coomassie blue stained gel of affinity purified wild-type (WT) and ΔAsp864_Gly885 proteins. **B** Immunoblot of wild-type (WT) and ΔAsp864_Gly885 proteins from ARPE-19 protein lysates. Vinculin (VCL) was used as loading control. **C**, **D** Quantitation showed that ABCA4 ΔAsp864_Gly885 expressed at levels similar to those measured for the WT ABCA4 in both HEK293T and ARPE-19 cells, respectively. Data are shown as mean±SEM. **E** ARPE-19 cells were transfected with the ABCA4 plasmids, and the localization of ABCA4 was detected using the ABCA4 3F4 clone (yellow). The ER, detected with anti-calnexin (CNX), is shown in magenta. ΔAsp864_Gly885 is associated with intracellular vesicles, that resembles the localization of the WT. Part of the truncated protein is retained in the ER, which was not identified with WT protein. Scale 20 μM. **Figure S3.** Percentages of *ABCA4 *Δexon 17 transcripts upon treatment with AONs in the first screening. The AONs were administered to WERI-Rb-1 cells gymnotically and the treatment lasted 48 h. Data are shown as mean±SEM, **p≤0.01, ****p≤0.0001 vs. untreated. **Figure S4**. Binding location of antisense oligonucleotides in first and second screening. Capital letters display *ABCA4 *exon 17. Colored horizontal lines represent the binding sites for RNA-splicing proteins (adapted from Alamut Visual Plus). The AONs in red represent the best candidates that were used in treatment of retinal organoids. **Figure S5.** The expression of retinal markers in retinal organoids (ROs) treated with AONs. All markers show similar expressions between all groups of ROs, except for *OPN1MW *that showed significant differences in ROs treated with AON24 and AON25 vs. scrambled group. Data are shown as mean±SEM. Asterisks indicated the significant differences in expression vs. scrambled, **p≤0.01, ***p≤0.001. **Table S1.** List of antisense oligonucleotides. **Table S2. **List of primers and probes used in dPCR assays.

**Additional file 2:** **Table S3. **Polymorphisms identified in the BA_13 plasmid upon mutagenesis.

**Additional file 3:** **Table S4. **SpliceAI predictions for the effect on splicing of canonical splice site variants.

**Additional file 4:** **Table S5. **The canonical splice site variants predicted to lead to complete exon skip with reported variants in *trans*.

**Additional file 5:** **Table S6. **The in-frame exon localization and role within the ABCA4 protein.

**Additional file 6:** **Table S7. **Data analysis for pathogenicity and severity allocation.

**Additional file 7:** **Table S8. ***ABCA4 *exon 17 variants.

**Additional file 8:** **Table S9. **In silico analysis of possible intergenic, intronic and coding off-targets with maximum of two mismatches in AONs used in retinal organoid treatment.

## Data Availability

All data generated throughout this study are available within the paper and its supplemental information. Raw data are available upon request from the corresponding author.

## Abbreviations

AAV: Adeno-associated virus
ABCA4: ATP-binding cassette, subfamily A, member 4
AON: Antisense oligonucleotide
cryo-EM: Cryogenic electron microscopy
CSS: Canonical splice site
ECD: Extracellular domain
FA: Fluorescein angiography
FAF: Fundus autofluorescence
ffERG: Full field electroretinography
LCA10: Leber congenital amaurosis type 10
NBD: Nucleotide-binding domain
N-ret-PE: N-retinylidene-phosphatidylethanolamine
RO: Retinal organoid
RP: Retinitis pigmentosa
SAS: Splice acceptor site
SD-OCT: Spectral domain optical coherence tomography
SDS: Splice donor site
STGD1: Stargardt disease type 1
TMD: Transmembrane domain
