# Supplementary material for: Stargardt disease-associated in-frame ABCA4 exon 17 skipping results in significant ABCA4 function
Source: J Transl Med. 2023 Aug 16;21:546. doi: 10.1186/s12967-023-04406-x (PMC10428568; doi:10.1186/s12967-023-04406-x)
Supplement: Supplementary file 1 — Additional file 1: Figure S1. Proband’s baseline full field electroretinography (ffERG) results (ISCEV protocol; MetroVision, Perenchies, France). A Moderately subnormal scotopic rod (dark adapted) responses for both left (OS, oculus sinister) and right eyes (OD, oculus dexter). B Moderately subnormal mixed response (dark adapted), of which the right eye shows a more diminished response. C Subnormal oscillatory responses (dark adapted) for both eyes (OP, oscillatory potential). D Very diminished cone responses (light adapted) in both eyes. E Subnormal flicker response (light adapted) for both eyes. Figure S2. Expression of ABCA4 constructs in HEK293T (A, C) and ARPE-19 (B, D, E) cells. A Coomassie blue stained gel of affinity purified wild-type (WT) and ΔAsp864_Gly885 proteins. B Immunoblot of wild-type (WT) and ΔAsp864_Gly885 proteins from ARPE-19 protein lysates. Vinculin (VCL) was used as loading control. C, D Quantitation showed that ABCA4 ΔAsp864_Gly885 expressed at levels similar to those measured for the WT ABCA4 in both HEK293T and ARPE-19 cells, respectively. Data are shown as mean±SEM. E ARPE-19 cells were transfected with the ABCA4 plasmids, and the localization of ABCA4 was detected using the ABCA4 3F4 clone (yellow). The ER, detected with anti-calnexin (CNX), is shown in magenta. ΔAsp864_Gly885 is associated with intracellular vesicles, that resembles the localization of the WT. Part of the truncated protein is retained in the ER, which was not identified with WT protein. Scale 20 μM. Figure S3. Percentages of ABCA4 Δexon 17 transcripts upon treatment with AONs in the first screening. The AONs were administered to WERI-Rb-1 cells gymnotically and the treatment lasted 48 h. Data are shown as mean±SEM, **p≤0.01, ****p≤0.0001 vs. untreated. Figure S4. Binding location of antisense oligonucleotides in first and second screening. Capital letters display ABCA4 exon 17. Colored horizontal lines represent the binding sites for RNA-splicing proteins (adapted fro [file 12967_2023_4406_MOESM1_ESM.pdf]

SUPPLEMENTARY DATA

Stargardt disease-associated in-frame *ABCA4* exon 17 skipping results in significant ABCA4 function

Melita Kaltak, Rocio Blanco-Garavito, Laurie L. Molday, Claire-Marie Dhaenens, Eric E. Souied, Gerard Platenburg, Jim Swildens, Robert S. Molday, and Frans P.M. Cremers

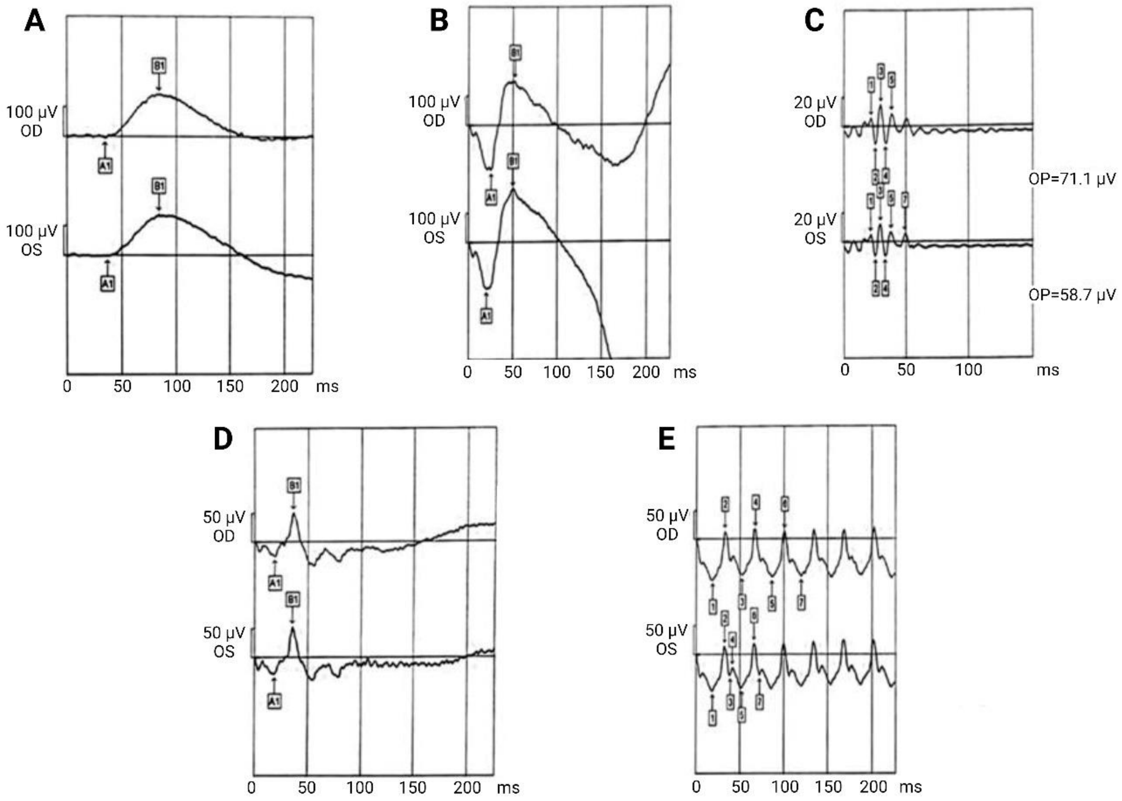

Figure S1. Proband's baseline full field electroretinography (ffERG) results (ISCEV protocol; MetroVision, Perenchies, France). (A) Moderately subnormal scotopic rod (dark adapted) responses for both left (OS, oculus sinister) and right eyes (OD, oculus dexter). (B) Moderately subnormal mixed response (dark adapted), of which the right eye shows a more diminished response. (C) Subnormal oscillatory responses (dark adapted) for both eyes (OP, oscillatory potential). (D) Very diminished cone responses (light adapted) in both eyes. (E) Subnormal flicker response (light adapted) for both eyes.

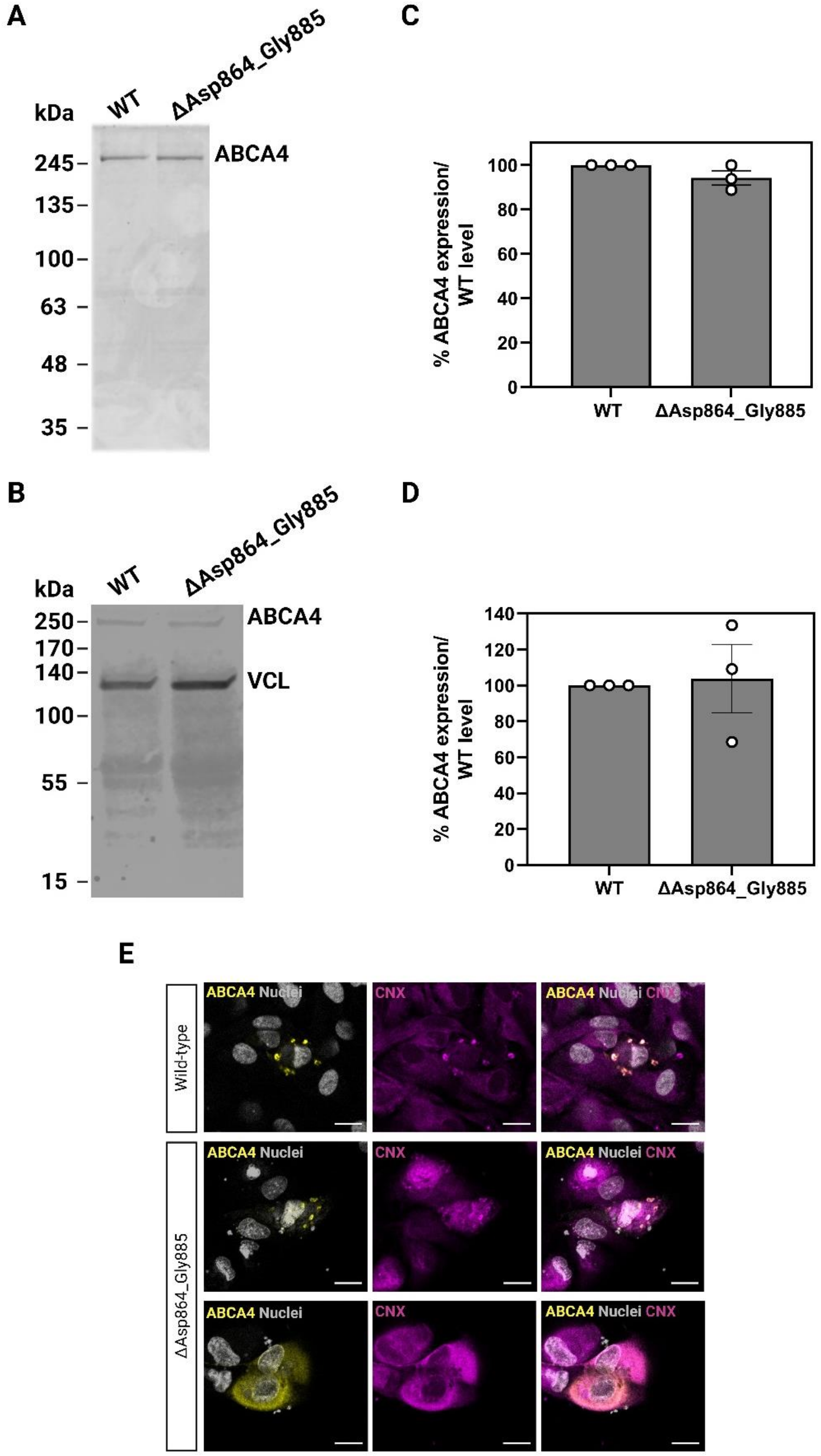

Figure S2. Expression of *ABCA4* constructs in HEK293T (A, C) and ARPE-19 (B, D, E) cells. (A) Coomassie blue stained gel of affinity purified wild-type (WT) and ΔAsp864_Gly885 proteins. (B) Immunoblot of wild-type (WT) and ΔAsp864_Gly885 proteins from ARPE-19 protein lysates. Vinculin (VCL) was used as loading control. (C, D) Quantitation showed that ABCA4 ΔAsp864_Gly885 expressed at levels similar to those measured for the WT ABCA4

in both HEK293T and ARPE-19 cells, respectively. Data are shown as mean±SEM. (E) ARPE-19 cells were transfected with the ABCA4 plasmids, and the localization of ABCA4 was detected using the ABCA4 3F4 clone (yellow). The ER was detected with anti-calnexin (CNX) showed in magenta. ΔAsp864_Gly885 is associated with intracellular vesicles, that resembles the localization of the WT. Part of the truncated protein is retained in the ER, which was not identified with WT protein. Scale 20 μM.

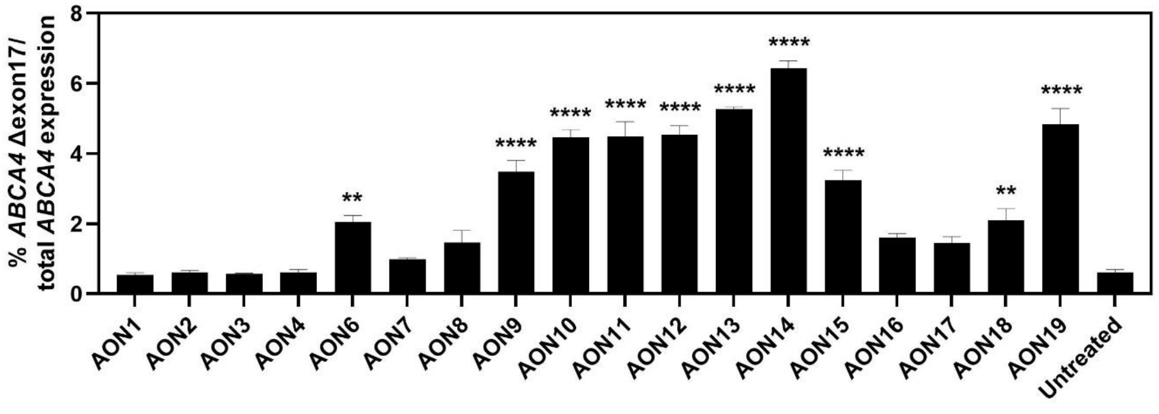

Figure S3. Percentages of *ABCA4* Δexon 17 transcripts upon treatment with AONs in the first screening. The AONs were administered to WERI-Rb-1 cells gymnotically and the treatment lasted 48h. Data are shown as mean±SEM, **p≤0.01, ****p≤0.0001 vs. untreated.

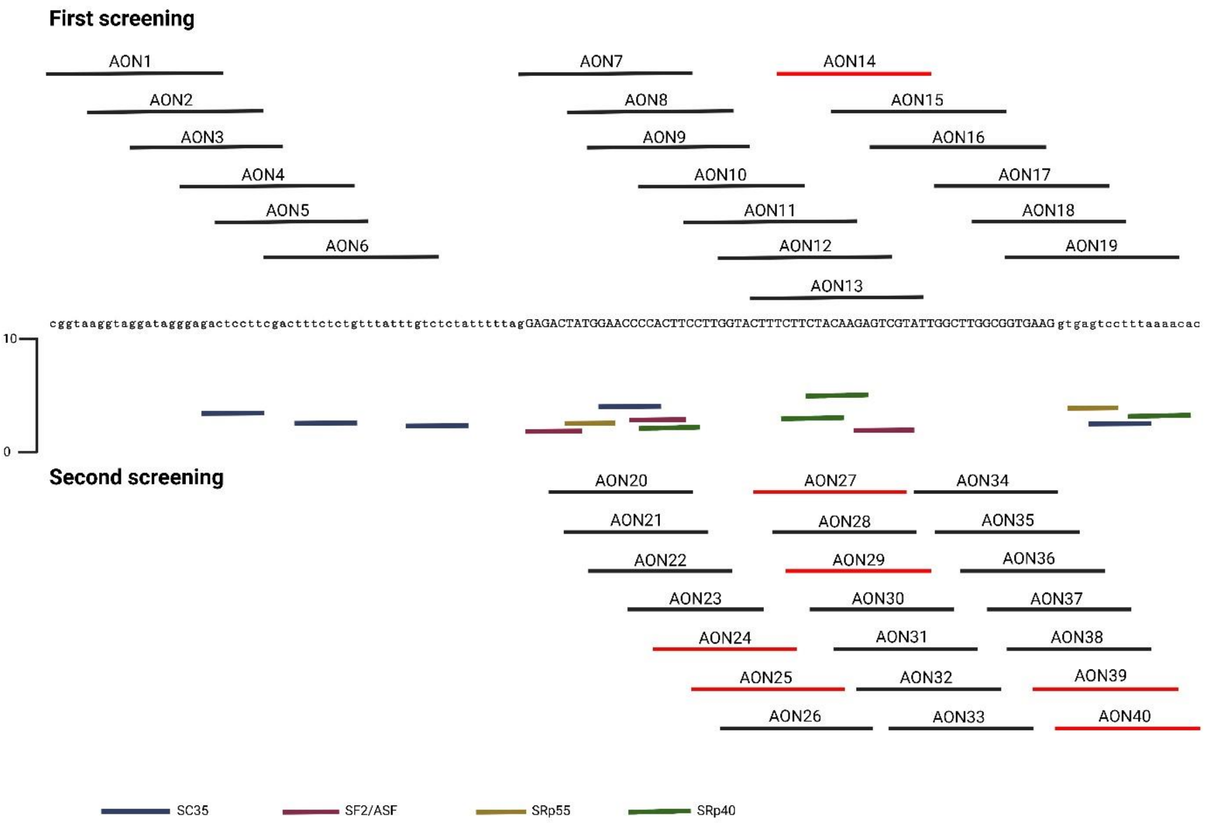

Figure S4. Binding location of antisense oligonucleotides in first and second screening. Capital letters display *ABCA4* exon 17. Colored horizontal lines represent the binding sites for RNA-splicing proteins (adapted from Alamut Visual Plus). The AONs in red represent the best candidates that were used in treatment of retinal organoids.

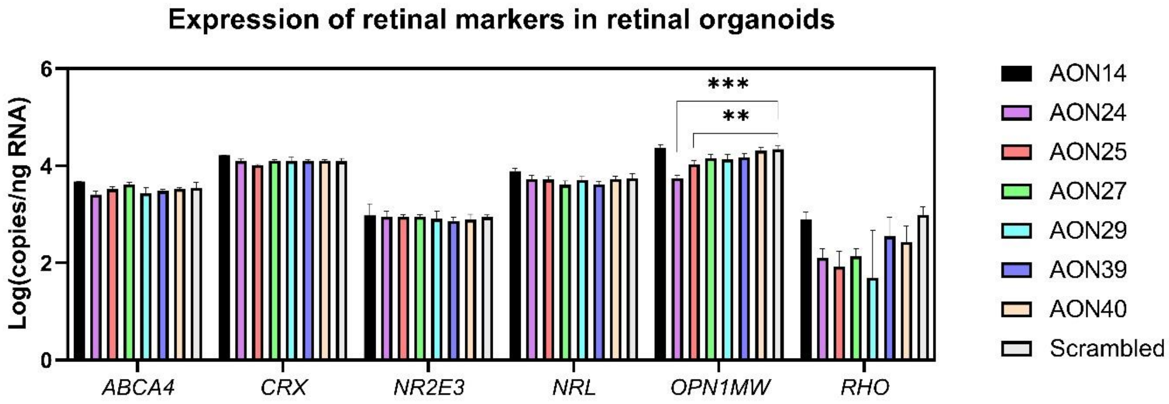

Figure S5. The expression of retinal markers in retinal organoids (ROs) treated with AONs. All markers show similar expressions between all groups of ROs, except for *OPN1MW* that showed significant differences in ROs treated with AON24 and AON25 vs. scrambled group. Data are shown as mean±SEM. Asterisks indicated the significant differences in expression vs. scrambled, **p≤0.01, ***p≤0.001.

Table S1. List of antisense oligonucleotides.

AON number	Sequence (5'- 3')	Start (GRCh37)	End (GRCh37)	Length (nt)
AON1	CGGTAAGGTAGGATAGGGAGAC	94517314	94517293	22
AON2	GGTAGGATAGGGAGACTCCTTC	94517308	94517287	22
AON3	GATAGGGAGACTCCTTCGAC	94517303	94517284	20
AON4	GAGACTCCTTCGACTTTCTCTG	94517297	94517276	22
AON5	CTCCTTCGACTTTCTCTGTT	94517293	94517274	20
AON6	CGACTTTCTCTGTTTATTTGTC	94517287	94517266	22
AON7	GGAGACTATGGAACCCCACTTC	94517255	94517234	22
AON8	CTATGGAACCCCACTTCCTTGG	94517250	94517229	22
AON9	GGAACCCCACTTCCTTGGTAC	94517246	94517226	21
AON10	CCACTTCCTTGGTACTTTCTTC	94517240	94517219	22
AON11	CCTTGGTACTTTCTTCTACAAG	94517234	94517213	22
AON12	GGTACTTTCTTCTACAAGAGTC	94517230	94517209	22
AON13	CTTTCTTCTACAAGAGTCGTAT	94517226	94517205	22
AON14	CTTCTACAAGAGTCGTATTGG	94517222	94517202	21
AON15	CAAGAGTCGTATTGGCTTGGCG	94517216	94517195	22
AON16	GTCGTATTGGCTTGGCGGTGAA	94517211	94517190	22
AON17	GGCTTGGCGGTGAAGGTGAGTC	94517203	94517182	22
AON18	GGCGGTGAAGGTGAGTCCTTT	94517198	94517178	21
AON19	GTGAAGGTGAGTCCTTTAAAAC	94517194	94517173	22
AON20	GACTATGGAACCCCACTT	94517252	94517235	18
AON21	CTATGGAACCCCACTTCC	94517250	94517233	18
AON22	TGGAACCCCACTTCCTTG	94517247	94517230	18
AON23	CCCCACTTCCTTGGTAC	94517242	94517226	17
AON24	CACTTCCTTGGTACTTTC	94517239	94517222	18
AON25	CCTTGGTACTTTCTTCTAC	94517234	94517216	19
AON26	GGTACTTTCTTCTACAAGA	94517203	94517212	19
AON27	CTTTCTTCTACAAGAGTCG	94517226	94517208	19
AON28	TTCTTCTACAAGAGTCGT	94517224	94517207	18
AON29	CTTCTACAAGAGTCGTAT	94517222	94517205	18
AON30	CTACAAGAGTCGTATTGG	94517219	94517202	18

AON31	CAAGAGTCGTATTGGCTT	94517216	94517199	18
AON32	GAGTCGTATTGGCTTGGC	94517213	94517196	18
AON33	CGTATTGGCTTGGCGGTG	94517209	94517192	18
AON34	ATTGGCTTGGCGGTGAAG	94517206	94517189	18
AON35	GGCTTGGCGGTGAAGGTG	94517203	94517186	18
AON36	TTGGCGGTGAAGGTGAGT	94517200	94517183	18
AON37	GCGGTGAAGGTGAGTCCT	94517197	94517180	18
AON38	GTGAAGGTGAGTCCTTTA	94517194	94517177	18
AON39	AAGGTGAGTCCTTTAAAAC	94517191	94517174	18
AON40	GTGAGTCCTTTAAAACAC	94517188	94517171	18

Table S2. List of primers and probes used in dPCR assays.

Target	Sequence (5'- 3')
ABCA4 junction exons 8-9 (total *ABCA4*)	CCCTCATGCAGAATGGTGGT (FW)
	CGCCCTCCAAGCGATTTTG (RV)
	/5HEX/AGAAGAACA/ZEN/ACATCCTTTTG/3IABkFQ/
ABCA4 junction exons 16-18 (Δexon 17)	GTGGAGCAACATCGGGAACA (FW)
	TGTTAGGGGCTCGGTCTTTT (RV)
	/56-FAM/TGTTTCCAG/ZEN/GGTGTTCAACC/3IABkFQ/
CRX	TTTGCCAAGACCCAGTACCC (FW)
	TTTAGCCCTCCGGTTCTTGA (RV)
	56-FAM/ATGCCCGTG/ZEN/AGGAGGTGGCT/3IABkFQ/
OPN1MW	GCCACCCTATGTGTGTCC (FW)
	GCTGTCCACACAGCAGCC (RV)
	/5HEX/AGAAGGCAA/ZEN/TGCCCACGATGGC/3IABkF
RHO	TCATGATGAACAAGCAGTTCC (FW)
	GTCTTGGACACGGTAGCAGA (RV)
	/5HEX/CATCTGCTG/ZEN/CGGCAAGAACCC/3IABkFQ/

Table S3. The Excel file “Table S3” contains polymorphisms identified in the BA_13 plasmid upon mutagenesis.

Table S4. The Excel file "Table S4" contains SpliceAI predictions for the effect on splicing of canonical splice site variants.

Table S5. The Excel file “Table S5” contains the canonical splice site variants predicted to lead to complete exon skip with reported variants in *trans*.

Table S6. The Excel file “Table S6” contains the in-frame exon localization and role within the ABCA4 protein.

Table S7. The Excel file “Table S7” contains Data analysis for pathogenicity and severity allocation.

Table S8. The Excel file “Table S8” contains *ABCA4* exon 17 variants.

Table S9. The Excel file "Table S9" contains *in silico* analysis of possible intergenic, intronic and coding off-targets with maximum of two mismatches in AONs used in organoid treatment.
